# ATP-Driven Contraction of Phage T3 Capsids with DNA Incompletely Packaged In Vivo

**DOI:** 10.3390/v9050119

**Published:** 2017-05-19

**Authors:** Philip Serwer, Elena T. Wright

**Affiliations:** Department of Biochemistry and Structural Biology, The University of Texas Health Science Center, San Antonio, TX 78229-3900, USA; wright@uthscsa.edu

**Keywords:** agarose gel electrophoresis, bacteriophage assembly, biological motor, hydration-based buoyant density fractionation, electron microscopy

## Abstract

Adenosine triphosphate (ATP) cleavage powers packaging of a double-stranded DNA (dsDNA) molecule in a pre-assembled capsid of phages that include T3. Several observations constitute a challenge to the conventional view that the shell of the capsid is energetically inert during packaging. Here, we test this challenge by analyzing the in vitro effects of ATP on the shells of capsids generated by DNA packaging in vivo. These capsids retain incompletely packaged DNA (ipDNA) and are called ipDNA-capsids; the ipDNA-capsids are assumed to be products of premature genome maturation-cleavage. They were isolated via preparative Nycodenz buoyant density centrifugation. For some ipDNA-capsids, Nycodenz impermeability increases hydration and generates density so low that shell hyper-expansion must exist to accommodate associated water. Electron microscopy (EM) confirmed hyper-expansion and low permeability and revealed that 3.0 mM magnesium ATP (physiological concentration) causes contraction of hyper-expanded, low-permeability ipDNA-capsids to less than mature size; 5.0 mM magnesium ATP (border of supra-physiological concentration) or more disrupts them. Additionally, excess sodium ADP reverses 3.0 mM magnesium ATP-induced contraction and re-generates hyper-expansion. The Nycodenz impermeability implies assembly perfection that suggests selection for function in DNA packaging. These findings support the above challenge and can be explained via the assumption that T3 DNA packaging includes a back-up cycle of ATP-driven capsid contraction and hyper-expansion.

## 1. Introduction

**Figure 1:**
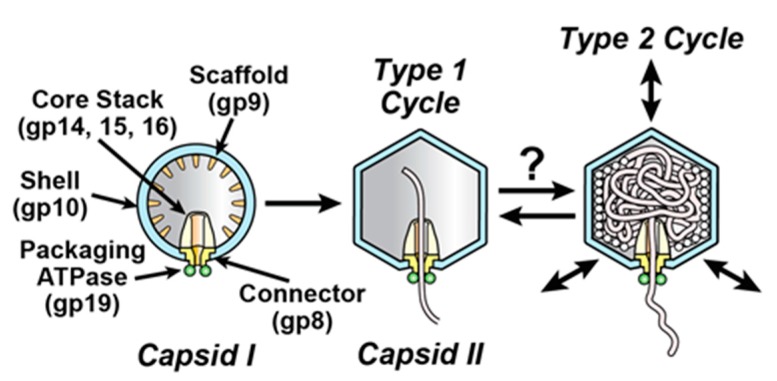
DNA packaging of the related phages, T3 and T7. DNA packaging in vivo is illustrated with capsid II participating in the type 1 and proposed type 2 cycles [2,6]. The connector/portal protein is gene product (gp) 8 (**yellow**); the packaging-promoting, ATP-cleaving enzyme (ATPase) is gp19 (**green circles**); the shell protein is gp10 (**blue**). The gp19 packaging ATPase is assumed to remain on capsid II during packaging, although gp19 has only been detected on capsid I, never on any form of capsid II. The assumption is that capsid II-associated gp19 dissociates from capsid II during capsid purification. The figure is modified from [2].

Double-stranded DNA (dsDNA) phages package DNA in a pre-formed capsid called a procapsid (capsid I for the related phages, T3 and T7; Figure 1). This packaging requires cleavage of adenosine triphosphate (ATP). Thus, the protein complex involved is called a DNA packaging motor. Investigation of phage DNA packaging motors reveals (1) stages in evolution [1,2]; (2) potential sources of relatively simple and effective, capsid-based drug delivery vehicles [3]; (3) new virus protein-specific drug targets for those eukaryotic viruses known [1] to package DNA via phage-homologous proteins; and (4) mechanisms of other biological motors [4,5].

Analysis of DNA packaging motors traditionally assumes an inert capsid shell (inert shell model). For T3/T7, this shell is an icosahedral assembly of protein gene product (gp) 10. The current analysis of energetics focuses on an ATP-cleaving enzyme (ATPase) attached to the external side of a connector/portal at the packaging vertex of the shell (green in Figure 1) [2,6,7,8,9]. In the case of T3/T7 and most other dsDNA phages, this ATPase has a C-terminal nuclease domain that cleaves the packaged genome from a multi-genome concatemer; the T3/T7 concatemer is not shown in Figure 1. Thus, the connector-associated ATPase is usually called terminase [2,6,7,8,9].

Nonetheless, ATPase activity has also been detected for capsid-assembled T3 gp10 in a defined (i.e., purified) in vitro DNA packaging system Table 3 of [10]). Evidence also exists for ATPase activity of protease-processed (i.e., post-procapsid) shell protein of phage T4 [11]. In addition, a minor amount of assembled T3 gp10 binds to an ATP analog [10]. These observations, coupled with an evolution-based plausibility analysis [2], suggest the conclusion that phage shells contribute to the ATP fueling of phage DNA packaging motors. A possible shell ATPase-specific function is removal of accidentally packaged non-DNA molecules, as suggested by the following: non-observation of protein or RNA co-packaged with phage DNA, despite packaging-associated holes that allow scaffolding proteins (e.g., gp9; Figure 1) to escape [2]. Finally, (1) shell flexibility-derived feedback to DNA packaging has been proposed to explain non-uniformity of a phage phi29 in vitro power stroke [12] and (2) flexibility of shell protein tertiary structure is indicated by the observation that packaging vertex-proximal shell protein subunits change conformation during binding to the packaging vertex of a phage phi29 DNA packaging-assisting RNA [13].

**Figure 2:**
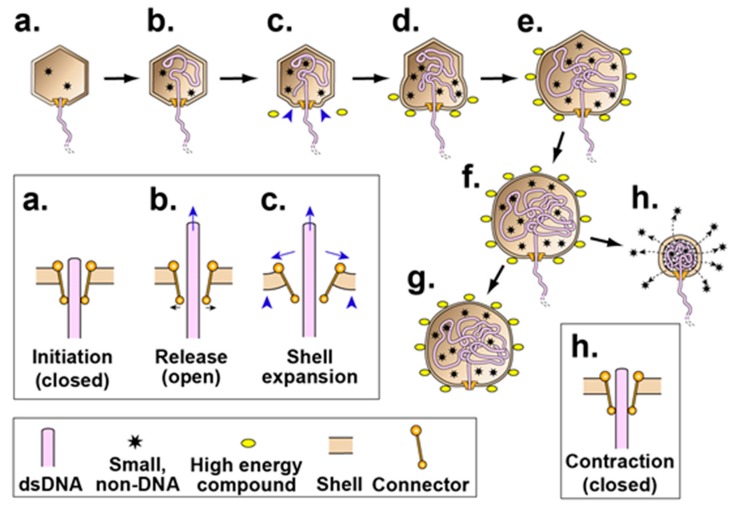
A version of the type 2 cycle, as proposed in reference 2 to exist before evolution of terminases. The legend at the bottom indicates the representation of a double stranded DNA (dsDNA) molecule, high-energy compound, packaged non-DNA molecule and capsid proteins. The insets (enclosed by rectangles) show the connector region of the corresponding capsid at higher magnification. (**a**,**b**) After initiation, opening of the connector releases the DNA molecule and (**c**) initiates shell expansion; (**d**–**f**) shell expansion is progressively driven by binding the high-energy compound, ATP (**yellow ovals with dark rim**). This binding is accompanied by shell permeability decrease to minimize packaging of non-DNA molecules. A source of the driving force for packaging is an osmotic pressure gradient across the shell produced by the shell expansion, coupled with impermeability of the shell to molecules in the bacterial cytoplasm. Relatively early in evolutionary time, the process of (**a**–**f**) ends with (**g**) external DNA subsequently digested. Subsequently, by this hypothesis, packaging evolves to be cyclic via (**h**) contraction of the shell, accompanied by opening of holes in the shell and expulsion of non-DNA molecules (stars) through these holes. In a new cycle, (**a**–**f**,**h**) repeat.

To account for the above observations, one hypothesis (type 2 cycle hypothesis) is that an anciently derived, ATP-fueled shell contraction/expansion cycle (type 2 cycle) backs-up the better studied, more recently evolved terminase cycle (type 1 cycle) of phage DNA packaging motors [2]. By this hypothesis, the type 2 cycle activates when the type 1 cycle stalls. Stalls typically occur because of the accidental packaging of non-DNA molecules, such as proteins and RNAs. The type 2 cycle then pumps non-DNA molecules out of the capsid while using connector-clamping to prevent expulsion of the DNA being packaged. A previously proposed [2], ancient (pre-terminase evolution) type 2 cycle is illustrated in Figure 2. 

In a subsequent study, the type 2 cycle hypothesis is found to correctly predict that T3 capsid II (Figure 1) is diverted to states of both hyper-expansion and contraction by osmotically shrinking the *Escherichia coli* host and, therefore, increasing intracellular concentration of protein and RNA molecules. A T3 mutant adapted to the shrunken host was used [14].

In this study, hyper-expanded capsid II is first detected during fractionation of capsid II by buoyant density centrifugation in a Nycodenz (Accurate Chemical and Scientific Corp., Brea, CA, USA) (molecular mass = 821) density gradient. Some wild type (WT) and mutant capsid II particles are Nycodenz-impermeable, which causes these capsids to behave as bags of water. The result is relatively low densities (Nycodenz low-density, or NLD, capsid II). The mutant NLD capsid II is less dense than the WT NLD capsid II and floats to the very top of a Nycodenz density gradient, where it is unaccompanied by detected host proteins. The density of the mutant NLD capsid II is less than 1.06 g/mL. A simple calculation shows that this density implies hyper-expansion in relation to WT NLD capsid II [14]. Electron microscopy (EM) confirms the hyper-expansion and reveals some contracted capsid II [14]. Unfortunately, most of this DNA-free, mutant NLD capsid II is aggregated.

The above data constitute a challenge to the predominant [2,6,7,8,9], inert shell model. Thus, in the current study, we tested the inert shell model against ATP-fueled dynamic shell models, such as type 2 cycle hypothesis. To do this, we (1) purified in vivo DNA packaging-generated, incompletely packaged DNA (ipDNA)-containing NLD capsid II (ipDNA-NLD capsid II) that is in a hyper-expanded, unaggregated state; and (2) tested the effects of ATP on these ipDNA-capsids. We observe dramatic effects of ATP, thereby supporting the above challenge.

## 2. Materials and Methods 

### 2.1. Propagation of Phages and Preparation of Phage-Infected Cell Lysates

Our 3-site T3 mutant, T3^SR3−^1 [15], was used to produce all ipDNA-capsids. The reason is that this mutant produced 5–10× more of the ipDNA-capsids observed here than did WT T3 when both were propagated in 2xLB medium: 20 g Bacto tryptone, 10 g Bacto yeast extract per liter, 0.1 M NaCl. T3^SR3−1^ had been selected in stages to propagate in 0.8 M NaCl-supplemented 2xLB medium. WT T3 did not propagate in the NaCl-supplemented medium. The host bacterium, *E. coli* BB/1, propagated slowly [15]. We prepared stocks of T3^SR3−1^ in the 0.8 M NaCl-supplemented 2xLB medium. We then used these stocks to generate ipDNA-capsids by infecting cells in 2xLB medium.

Phage infectivity titers were obtained via conventional plaque assays at 30 °C. T3^SR3−1^ had three point mutations in tail genes (genes 11 and 12), one in an exonuclease gene (gene 6) and one in a gene for an early protein of unknown function (gene 1.5). These mutations were detected by whole genome sequencing [15].

To generate either a T3^SR3−1^- or a WT T3-infected cell lysate, an aerated, six-liter, log phase culture of host cells in 2xLB medium was grown to 3.0 × 10^8^ per mL. This culture was infected at a multiplicity of 0.01. Incubation was continued until lysis at ~120 min.

We purified WT phage T3 in cesium chloride density gradients, using procedures previously described [16]. We purified WT NLD capsid II using procedures previously described, finishing with buoyant density centrifugation in a Nycodenz density gradient [3].

### 2.2. Fractionation of ipDNA-Capsids

Preparation of a lysate for fractionation was performed by (1) addition of 50 g/L of NaCl; (2) clarification via low speed pelleting; (3) double precipitation of capsid-sized particles with polyethylene glycol and (3) reduction of viscosity by low-concentration DNase I digestion, all as previously described [15].

The concentrated, clarified particles, including ipDNA-capsids, were fractionated, first, by cesium chloride step gradient ultracentrifugation and, second, by cesium chloride buoyant density ultracentrifugation [15,16]. Before further fractionation, ipDNA-capsids were dialyzed against Tris-Mg buffer: 0.1 M NaCl, 0.01 M Tris-Cl, pH 7.4, 0.001 M MgCl_2_.

The final fractionation step was buoyant density centrifugation in a Nycodenz density gradient. Particles from the second cesium chloride gradient were dialyzed against Tris-Mg buffer and then were added to a solution of Nycodenz in Tris-Mg buffer: final volume = 2.0 mL; final density, ρ = 1.172 g/mL. The ρ was determined by measuring the refractive index, η, at 20 °C: ρ = 3.242η − 3.323 [17]. This 2.0 mL solution was (1) placed in a 5 mL Beckman Ultra-Clear centrifuge tube and overlaid with 3.2 mL of mineral oil (Mallinkrodt Pharmaceuticals, Dublin, IE; product #6358); and (2) centrifuged in a Beckman SW55 rotor (Beckman LE80 ultracentrifuge; Beckman Coulter Life Sciences, Indianapolis, IN, USA) for 18 h, at 42,000 rpm, 10 °C. We collected fractions via tube puncture, from the bottom of the centrifuge tube, through an 18-gauge needle. To avoid viscosity-promoted mixing during collection, we used a low collection rate, <1 drop per 1.5 s. The densities of all fractions were measured by determining the refractive index within 0.5 h of fraction collection.

### 2.3. Gel Electrophoresis of DNA: Detection of ipDNA-Capsids

To expel packaged DNA before gel electrophoresis, we added the following to a 12 µL portion of an undialyzed fraction of a Nycodenz density gradient: (1) 5 µL of 0.1 M NaCl, 0.01 M Tris-Cl, pH 7.4, 0.001 M ethylenediaminetetraacetic acid (EDTA) and (2) 3 µL of 30% sucrose, 0.6 M NaCl, 0.06 M Tris-Cl, pH 7.4, 0.06 M EDTA, 6% Sarkosyl NL97, 1.2 mg/mL bromophenol blue. This mixture was then incubated at 85 °C for 10 min. For gel electrophoresis, a portion of this mixture was layered in a sample well of a submerged 0.7% agarose (Seakem LE, Lonza Group, Rockland, ME, USA) gel. This gel had been cast in and submerged under 0.05 M sodium phosphate, pH 7.4, 0.001 M EDTA. We then performed electrophoresis in this buffer at 0.5 V/cm, 25 °C for 26.0 h. After electrophoresis, we stained nucleic acid in the gel by soaking the gel for 2 h. at room temperature (25 ± 2 °C) in 1:10,000 diluted GelStar (New England Biolabs, Ipswich, MA, USA) in 0.002 M sodium EDTA, pH 7.4. We destained the gel in 0.002 M sodium EDTA, pH 7.4 for at least 2 h. We photographed the gel during top-illumination with a Fotodyne #3-3000 transilluminator.

### 2.4. SDSPAGE

The procedure for sodium dodecyl sulfate polyacrylamide gel electrophoresis (SDS-PAGE) has been previously described [14,15]. A 9% gel was used with silver staining via the Bio-Rad Silver Stain #161-4443 (Hercules, CA, USA), used according to the manufacturer’s instructions.

### 2.5. Electron Microscopy: Incubation with ATP and ADP

For negative staining, a sample from a Nycodenz density gradient was (1) adsorbed to a carbon support film for 5 min; (2) washed with 3–5 drops of 0.2 M NaCl, 0.01 M Tris-Cl, pH 7.4, 0.001 M MgCl_2_ and then (3) stained with 3 drops of 1% sodium phosphotungstate, pH 8.4. The excess stain was removed by wicking with Whatman filter paper #1 (Cole-Parmer, Vernon Hills, IL, USA). The sample was air-dried and placed in an electron microscope within an hour of preparation. To expose ipDNA-capsids to ATP or ADP before electron microscopy, a 0.2 M solution of ATP or ADP was diluted in Tris-Mg buffer and, then, further diluted into a portion of a Nycodenz fraction at the indicated final ATP or ADP concentration. As indicated, the ATP and ADP had been dissolved and neutralized with either magnesium hydroxide (magnesium ATP/magnesium ADP) or sodium hydroxide (sodium ATP). Negatively stained specimens were observed with a JEOL100CX electron microscope in the Department of Pathology at the University of Texas Health Science Center at San Antonio.

The carbon support films were made extra-adherent by preparation between two parlodion (Fullam, Inc., Latham, NY, USA) films: parlodion-smooth carbon-fenestrated carbon-parlodion. The procedure has been previously described [18]. At 20–40 min before preparing a specimen, the outer, parlodion layers were dissolved by washing with amyl acetate, followed by acetone. The washed support film was air-dried for at least 10 min before use. After washing, the fresh carbon surface was more capsid-adherent than a carbon film prepared without parlodion layers.

## 3. Results

### 3.1. Fractionation of ipDNA-Capsids

For reasons not known, T3^SR3−1^ produced ipDNA-capsids in greater amount and stability than did WT T3. T3^SR3−1^ ipDNA-capsids were initially fractionated by (1) concentration from a lysate via polyethylene glycol precipitation; (2) centrifugation into a cesium chloride step gradient and (3) buoyant density centrifugation in a cesium chloride density gradient of a broad ipDNA-capsid fraction from the step gradient [16]. In step (3), the ipDNA-capsids were fractionated by length of ipDNA [16], which we will represent as the fraction (*F*) of the length of mature T3 DNA. This latter length is 38.202 Kb [19].

**Figure 3:**
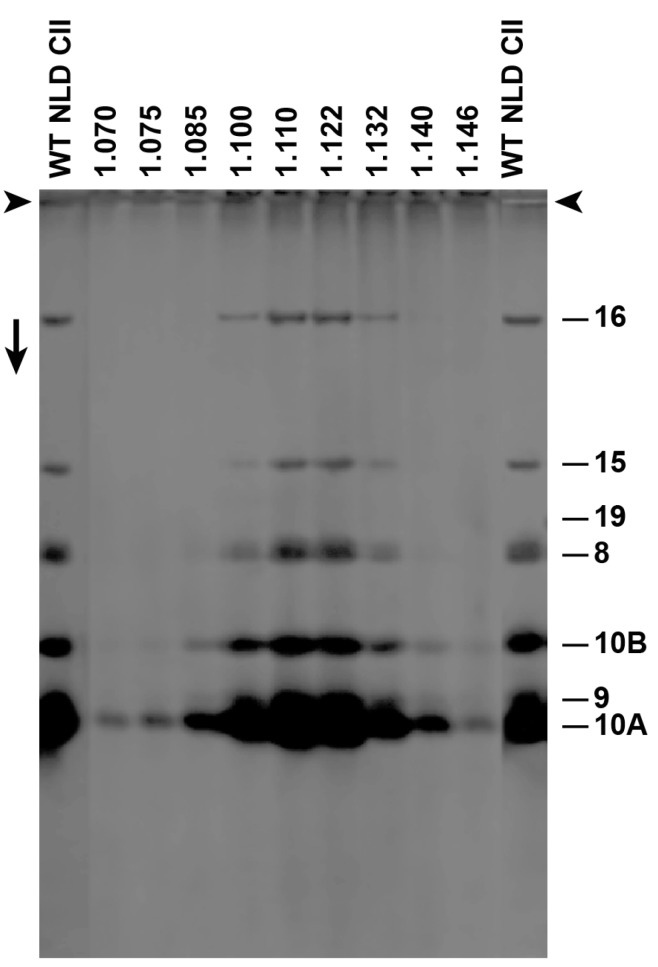
Sodium dodecyl sulfate polyacrylamide gel electrophoresis (SDS-PAGE) of Nycodenz low-density (NLD) incompletely packaged DNA (ipDNA)-capsids (*F* = 0.27–0.31). Densities (g/mL) of Nycodenz fractions are above lanes. Number of protein [19] is at the right. WT NLD CII = wild type NLD capsid II; arrowheads indicate origins; the arrow indicates electrophoretic direction.

We subjected *F*-fractionated ipDNA-capsids to buoyant density centrifugation in a Nycodenz density gradient. For ipDNA-capsids with each of several *F* values, this procedure separated Nycodenz-impermeable, NLD ipDNA-capsids from Nycodenz-permeable, high-density (NHD) ipDNA-capsids. The NLD ipDNA-capsids were also separated from components of the host cell, such as outer membrane vesicles. We tested for non-capsid proteins by SDS-PAGE and did not detect any non-capsid proteins in NLD ipDNA-capsids (Figure 3). The protein composition was that of capsid II; no gp19 was observed with a sensitivity limit of about three gp19 molecules per capsid. The missing host proteins included ompA and ompF, previously found to be the major proteins of outer membrane vesicles in phage T7 capsid preparations that did not include a Nycodenz-density separation [20]. The density shift from cesium chloride to Nycodenz density gradients was sufficient to effectively eliminate host vesicles from the preparations observed by the EM, below.

**Figure 4:**
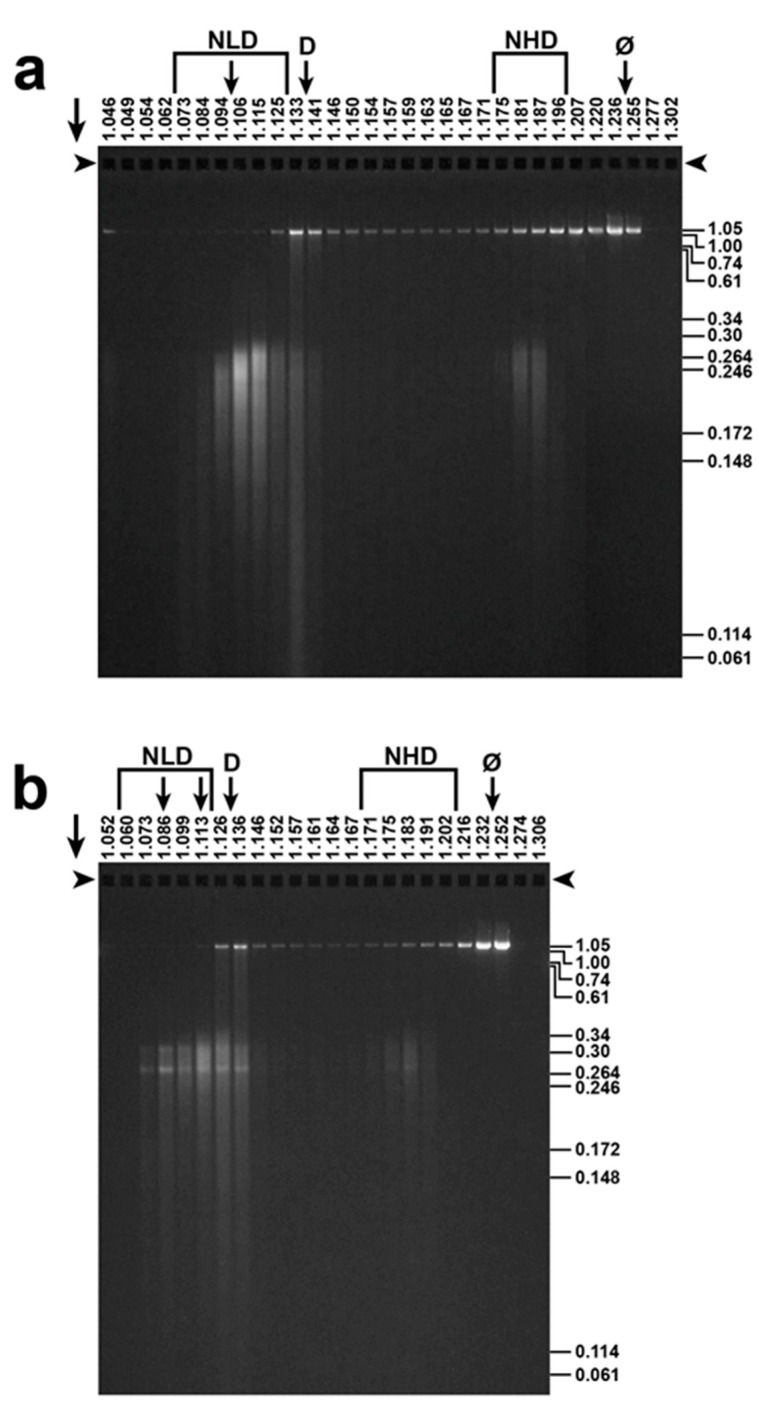
Final fractionation and detection of T3 ipDNA-capsids. After buoyant density centrifugation in a Nycodenz density gradient, the ipDNA of ipDNA-capsids with peak *F* values of (**a**) 0.23–0.27 and (**b**) 0.27–0.30 was expelled and was analyzed by agarose gel electrophoresis. *F* values, indicated at the right, were determined via co-electrophoresis of the length standards of [15]. Arrows above an arrowhead indicate electrophoretic direction; arrowheads indicate origins; NLD: Nycodenz low density; NHD: Nycodenz high density; D: free DNA, Ø: Infective phage

The ipDNA-capsids were initially detected by gel electrophoresis of the ipDNA after (1) DNase I digestion of DNA not packaged and (2) DNA expulsion from capsids [15]. Figure 4 shows this analysis (DNase I protection analysis) for fractions of Nycodenz density gradients that had fractionated ipDNA-capsids with peak *F* values of 0.23–0.27 (Figure 4a) and 0.27–0.31 (Figure 4b). Nycodenz density (g/mL) is at the top of a lane; *F* is at the right, based on migration, not shown in the figure, of DNA standards. The *F*-value difference between Figure 4a,b is visually detectable. Indicators of low aggregation were (1) the fractionation by *F*, including additional fractionations at higher *F* values up to 0.95 (not shown); and (2) EM, below.

The intensity of ipDNA staining in the various lanes of Figure 4 revealed peaks of NLD and NHD ipDNA-capsids, as marked in Figure 4. This NLD–NHD separation was caused by a Nycodenz impermeability-generated high hydration of the NLD ipDNA-capsids, as previously shown for DNA-free capsids [14,21,22]. Vertical arrows under NLD in Figure 4b indicate sub-peaks within the NLD region; D indicates the position of protein-free DNA, identified by agarose gel electrophoresis; φ indicates the position of infective phage.

The lowest-density NLD ipDNA-capsids (leftmost vertical arrow under NLD in Figure 4b) were hyper-expanded (not a debatable point) because they had densities of 1.070–1.086 g/mL. These densities, as observed in the current conditions (densities vary slightly with hydrostatic pressure and use of either Nycodenz or Metrizamide), were lower than the density of either capsid-free DNA (1.134 g/mL; lowered by high bound-water hydration [23,24]) or the NLD form of WT capsid II [21,22] (1.112 g/mL). This was true even though the latter has been found by cryo-EM [25] to be 1.4% larger than the mature phage capsid. Precise calculation of hyper-expansion cannot be done because of the unknown DNA hydration, sensitive to position in the density gradient [23].

### 3.2. Electron Microscopy (EM)

**Figure 5:**
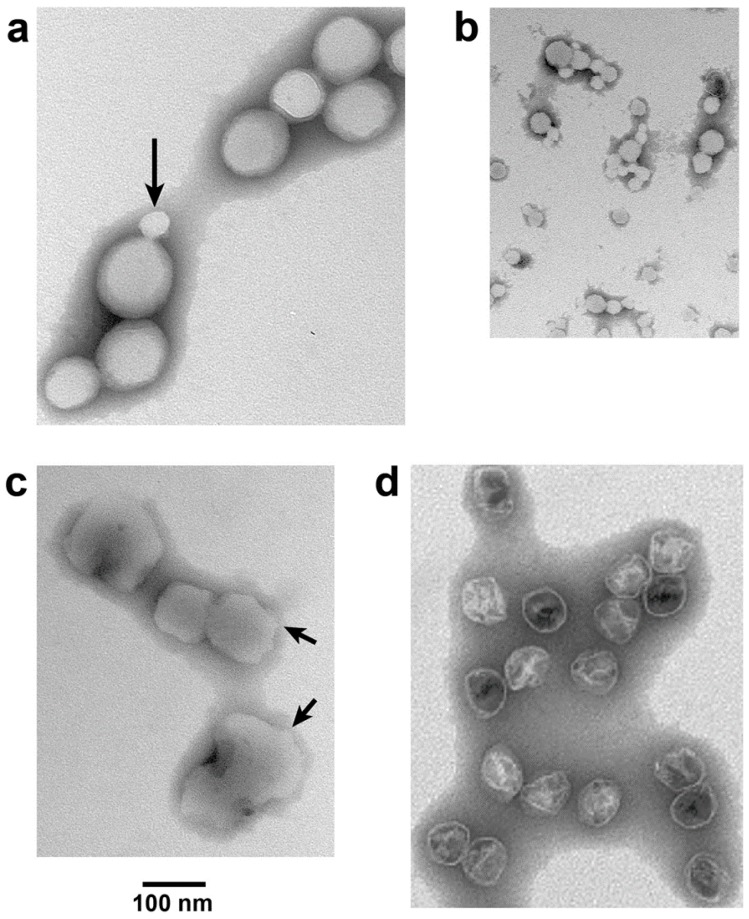
Response of ipDNA-capsids to ATP. Electron microscopy (EM) of (**a**) untreated, peak *F* = 0.30–0.41-NLD ipDNA-capsids, Nycodenz density = 1.070 g/mL; (**b**) the sample of (**a**) exposed to 3.0 mM magnesium ATP at 30 °C for 1.0 h; (**c**) the sample of (**b**) exposed to 100 mM sodium ADP at 30 °C for 1.0 h; (**d**) WT NLD capsid II. All samples were prepared for EM by negative staining. Images have equal magnification.

For NLD ipDNA-capsids that had the lowest Nycodenz density in both density gradients of Figure 4 (e.g., 1.086 g/mL fraction indicated by leftmost arrow under NLD in Figure 4b), EM revealed characteristics previously observed [14] for DNA-free, hyper-expanded NLD capsid II. The specimens were prepared by negative staining. Most (82.8%) *F* = 0.30–0.41-NLD ipDNA-capsids (Figure 5a) appeared 1.2–2.3× larger than WT NLD capsid II; the latter is shown in Figure 5d. Some (10.3%) appeared at least 15% smaller (arrow in Figure 5a). The latter particles usually had the angular appearance of an icosahedral protein shell (arrow in Figure 5a) and were assumed to be contracted capsid II, as supported below. In contrast, the denser NLD particles in Figure 4b (rightmost arrow under NLD, 1.113 g/mL) were more than 90% not hyper-expanded, as were the NHD ipDNA-capsid II particles; both appeared primarily contracted. Statistics, here and below, were based on 400–600 randomly selected particles.

The apparent hyper-expansion of Figure 5a was real for the following reasons. As generally true for negatively stained particles [26], the observed NLD ipDNA-capsid II particles had volume altered by dehydration. The dehydrated radius of the shell of the T7 phage capsid was 22–23 nm (unflattened) [26]. This was presumably the dehydrated radius for the almost identical [25] shell of T7 capsid II. T3 phage and T3 NLD capsid II have been found to be size-indistinguishable from their T7 counterparts [22]. Thus, in Figure 5a, particle flattening could not explain any apparent radius greater than 33 nm because, based on geometry, the maximum flattening-induced radius increase was (2)^1/2^× = 1.424×. This point was made especially dramatic by an ~1.2× flattening of T7 capsid I (and presumably WT NLD capsid II), as calculated from the results of tomography in reference 18.

Almost all (>99%) NLD ipDNA-capsids also had unusually high electron transparency (Figure 5a). Similar transparency was previously observed in DNA-free, hyper-expanded capsid II [14]. The transparency was caused by impermeability to the staining anion, phosphotungstate [14]. The remaining particles had the appearance of WT capsid II.

### 3.3. Effects of ATP on NLD ipDNA-Capsid II

Incubation at 30 °C in the presence, but not in the absence, of 3.0 mM ATP had a dramatic effect on hyper-expanded NLD ipDNA-capsid II. This concentration of ATP is in the range of physiological concentrations [27]. The NLD ipDNA-capsid II particles originally appeared 82.8% hyper-expanded, as in Figure 5a (10.3% contracted, 6.9% indistinguishable in radius from WT NLD capsid II). Incubation with 3.0 mM ATP yielded a different population (Figure 5b) that appeared 91.5% contracted to 0.37–0.80× WT NLD capsid II radius (2.9% hyper-expanded, 5.6% indistinguishable in radius from wild type NLD capsid II). This contraction effect was seen in three independent trials and was specific for the NLD ipDNA-capsids. WT NLD capsid II underwent no detected change during this incubation. Phage T3 underwent no change in titer. 

The contraction effect also was specific for physiological ATP concentration. When the concentration of magnesium ATP was raised to 5.0 mM (one trial), which is at the border of physiological and supraphysiological [27], at least 90% of capsids were replaced by smaller, more asymmetrical particles. These were assumed to be capsid fragments (not shown). At supra-physiological 10 and 100 mM magnesium ATP, no capsids were observed, only fragments (one trial, each). The ipDNA-capsids did not appear altered when 3.0 mM magnesium ADP was used instead of 3.0 mM ATP; the capsids did not appear fragmented when ADP concentration was raised to 100 mM (one trial).

### 3.4. Reversal of the ATP-Induced Contraction

The contraction effect was not caused by loss of subunits because this effect was reversed by subsequent incubation of magnesium ATP-contracted NLD ipDNA-capsids with 100 mM sodium ADP. Capsids appearing re-hyper-expanded (Figure 5c) were 93.3% of the total (2.2% contracted; 4.4% indistinguishable in radius from WT NLD capsid II). The particles in Figure 5c were (1) not as rounded as the originals in Figure 5a and (2) not as electron transparent; arrows in Figure 5c indicate relatively non-rounded zones. A potential reason is the non-physiological environment in which the expansion of Figure 5c occurred.

## 4. Discussion

### 4.1. Electron Microscopy (EM)

The objects visualized in Figure 5a–c are NLD ipDNA-capsids, based on composition and EM-similarity to DNA-free capsids observed in reference 14. However, the gp10 shells of these ipDNA-capsids do not have the appearance of most capsid shells previously observed. This, of course, means that these shells are different in some basic way. It does not mean that they are not the shells of T3 capsids. At least part of the difference is the impermeability. Thus, (1) a stain-filled cavity is not observed and (2) deceptively, the hyper-expanded versions in Figure 5a do not appear to have an interior more dilute than the contracted versions in Figure 5b.

If one were to use EM to discriminate hyper-expanded and contracted NLD ipDNA-capsids in a less purified preparation, then a criterion would be the impermeability to phosphotungstate, seen via high electron transparency. For example, outer membrane vesicles in T7 capsid preparations have an interior permeated by phosphotungstate [20]. These outer membrane vesicles also have a more variable shape, some having the shape of dumbbells [20]. 

### 4.2. DNA Packaging

The following are the key observations of our test of the inert shell model versus the ATP-fueled dynamic shell model. Hyper-expanded T3^SR3−1^ NLD ipDNA-capsids undergo contraction (1) induced by ATP at physiological concentration, but not by ADP at the same concentration; and (2) linked to the presence of ipDNA. Thus, the shell of NLD ipDNA-capsid II has the capacity to transduce energy from ATP. Indeed, T3 capsid-assembled gp10 has previously been shown [10] to have ATPase activity.

These observations obviously favor an ATP-fueled dynamic shell model of packaging if, in the present study, we are mimicking in vivo packaging. We are mimicking something that occurs in vivo because (1) NLD ipDNA-capsid II is generated by in vivo DNA packaging and (2) ipDNA-free NLD capsid II does not respond to ATP. Contraction starts and ends with a phosphotungstate-sealed gp10 shell. This latter observation supports a role in DNA packaging, as discussed in the next paragraph. These observations are novel for any virus, as far as we know.

Nonetheless, the question arises of whether the in vivo process being mimicked is the formation of dead-end products that do not progress to produce an infective phage particle. The likely answer is no and is embedded in the perfection of shell sealing for NLD ipDNA-capsid II. Based on the quasi-atomic, cryo-EM-based structure of WT NLD capsid II [25], this perfection requires the maintenance of a complex network of inter-gp10 subunit interactions, including non-covalent topological linking. Thus, the gp10 interactions of the NLD ipDNA-capsid II shell are likely to be the product of prolonged evolutionary selection. Thus, the shell states observed here are likely to be DNA packaging motor-generated precursors to infective phages in vivo. 

The type 2 cycle of Figure 2 is a concrete, illustrative example of what the ATP-driven shell activity might be. Further work is needed to determine what the details really are. Projected future work includes structure-based analysis of both isolated motors and motors while they are packaging DNA in an infected cell.

We note the following in anticipation of what might be found. Hyper-expansion requires a major change in gp10 conformation, probably including increased beta sheet [14]. We speculate that this change begins at the N-terminus because, during the T7 capsid I to capsid II transition, this region of gp10 undergoes (1) unraveling of an alpha helix and (2) movement from the inside to the outside of the gp10 shell [25]. Inspection of the N-terminal gp10 sequence [19] reveals no strict Walker A [GXXXXGK(T/S)] or universal stress protein [G-2X-G-9X-G(S/T)] [28] sub-sequence. The N-terminal sequence is MANIQGGQQIGTNQGKGQSAADKLALFLKVF. However, the N-terminal sequence does have a glycine-rich, near-Walker A sub-sequence (GGQQIGT). This sub-sequence is missing the Walker A (phosphate-binding) lysine and is underlined above. Thus, to provide detail to Figure 2, the proposal is that connector-initiation of hyper-expansion (Figure 2c) refolds the N-terminus of gp10 to generate an ATP binding site at GGQQIGT. Subsequent ATP binding to this site propagates the refolding and hyper-expansion (Figure 2d–f).

In this hypothetical scenario, our hyper-expanded NLD ipDNA-capsids have lost some or all ATP during our isolation of them, but have remained hyper-expanded. Upon regaining ATP in vitro in Figure 5b, the shell undergoes the contraction (illustrated in Figure 2f,h) that it was about to undergo before interruption by cellular lysis. The re-hyper-expansion in Figure 5d would, by this reasoning, be a non-physiological effect in which a relatively large concentration of ADP mimics the loss/re-binding of ATP.

### 4.3. Strategy

The unique observations of this study required isolation of NLD ipDNA-capsids. Isolation of NLD ipDNA-capsids requires the use of both hydration-based separation by buoyant density centrifugation and directed evolution-based genetics. This type of strategy also has the potential to accomplish the “de-obscuring” of the in vivo intermediate states of other biological motors.
